# International Perspectives on Adolescent and Young Adult Drinking

**Published:** 2004

**Authors:** Salme K. Ahlström, Esa L. Österberg

**Affiliations:** Salme K. Ahlström, Ph.D., is a research professor, and Esa L. Österberg, M.Sc., is a senior researcher, both in the Alcohol and Drug Research Group, National Research and Development Centre for Welfare and Health, Helsinki, Finland

**Keywords:** young adult, adolescents, AOD (alcohol and other drug) consumption, AOD use frequency, AOD intake per occasion, AOD use pattern, age of AODU (alcohol and other drug use) onset, heavy drinking, AOD abstinence, factors determining AOD demand, AOD effects and consequences, international AODR (alcohol and other drug related) problems, international aspects, ethnic differences, cultural patterns of drinking, gender differences, minimum drinking age laws, prevention through decreasing availability and accessibility, World Health Organization (WHO), European School Survey Project on Alcohol and Other Drugs (ESPAD)

## Abstract

Alcohol consumption by adolescents and young adults varies greatly in different countries and cultures, in different population groups within a country, and over time. Analyses of per capita consumption in different countries provide some information on drinking patterns of young people in various countries. School-based surveys conducted in a variety of European countries and in the United States offer more specific insight into the drinking behavior of this age group. Such surveys have analyzed variables such as age of onset of drinking; lifetime frequency of drinking; drinking to intoxication; frequency, amount, and timing of current drinking; and drinking consequences. These studies have demonstrated that drinking patterns of young people in, for example, Scandinavian, Anglo-Saxon, and Mediterranean countries vary greatly. Further analyses have explored the influence of social norms and related factors as well as alcohol availability and pricing on alcohol consumption among adolescents and young adults. The generalizability of the findings is limited, however, by the fact that most studies have been conducted in the United States and Europe.

Studies conducted in various countries have demonstrated that both the frequency of drinking alcoholic beverages and the amount of alcohol consumed per person or per occasion vary greatly among different countries and cultures, among different population groups within a given country, and for each population over time. Similarly, the rates of alcohol-related problems vary greatly among different countries and among different population groups. These differences are found not only for adult drinkers but also for adolescents and young adults.

One useful measure that can easily be determined in many countries is the total alcohol consumption of the population, which can be converted to average per capita consumption. This variable is related to the prevalence of heavy alcohol use and also is an important indicator of the prevalence of alcohol-related problems (Bruun et al. 1975; Edwards et al. 1994). The relationship between average per capita consumption and the level of alcohol-related problems in a population is influenced by the following factors:

- The number of drinkers in the population and their drinking habits (Babor et al. 2003). For example, if total alcohol consumption can be attributed to only 20 percent of the population, who mostly drink on the weekends (and the remaining population does not drink), the prevalence of heavy drinking and of alcohol-related problems will be different than if alcohol consumption can be attributed to 90 percent of the population who generally have only one drink per day.
- The drinking culture and attitudes toward drinking and alcohol-related problems. “Harmful” drinking and alcohol-related problems are in part culturally defined—that is, a behavior (e.g., drinking to intoxication) considered problematic in one culture may not be thought of as problematic in another culture.
- Overall historical, cultural, economic, and social circumstances that affect many areas related to alcohol consumption. For instance, if drinking habits are similar in two countries but people in one of those countries are significantly less likely to own a car, then the frequency of drunk driving and the proportion of alcohol-related deaths among all traffic fatalities will differ greatly between the two countries.
- Alcohol control measures and their enforcement. For instance, increases in the legal drinking age and effective enforcement of the new age limit will lead to lower alcohol consumption levels and fewer alcohol-related problems among young adults and adolescents.

The relationships between alcohol consumption and alcohol-related problems, as well as the factors that influence these relationships, apply to both adult and adolescent drinking. When trying to compare adolescent or young adult alcohol consumption across countries or cultures, however, researchers must keep in mind that the definitions of these two developmental stages can vary according to the cultural and historical background of the society under investigation. Broadly defined, adolescence and young adulthood include the period of transition from childhood to adulthood. It is the time when a person acquires the skills needed to cope with the emotional, physical, social, and economic separation from parents. Ideally, it also is the time when a person paves the way for establishing his or her own family, raising children, and participating in social and work life as well as leisure time activities as an independent individual. Because these developments may occur at different ages in different cultures, it is difficult to define adolescence and young adulthood in terms of exact ages. For practical purposes, in most Western industrialized societies, adolescence is defined as ages 14 through 18 and young adulthood as ages 19 through 25 (Ahlström 2000). In some studies, even 29-year-olds have been considered young adults (Rehm et al. 2001).

After a brief review of alcohol consumption patterns in the entire population (which to a certain extent also reflect consumption patterns by younger people), this article provides an international perspective on alcohol consumption among adolescents and young adults and examines gender differences, abstinence rates, the amount and frequency of drinking, as well as drinking to intoxication. This discussion considers not only the actual drinking patterns but also the drinking contexts (i.e., the time and place of drinking and the nature of the drinking occasion). It is important to keep in mind, however, that the information on drinking patterns usually comes from survey studies, which can differ greatly in how different aspects of drinking patterns are defined and measured (Simpura and Karlsson 2001). Furthermore, most of the available research data were obtained in the developed countries, which may limit the generalizability of these research findings to adolescent and young adult drinking in other areas of the world.

## International Comparisons of Adult Alcohol Consumption Patterns

Drinking alcohol is a social behavior in the sense that it is something young people learn and practice with other members of their culture (Edwards et al. 1994). Drinking patterns among adolescents and young adults in any country or culture consequently are related in many ways to the drinking patterns of the entire population (Room 2004). Therefore, a global review of per capita alcohol consumption and general drinking patterns also gives a first rough description of the differences in the amounts and patterns of adolescent and young adult drinking in different parts of the world. Adolescent and young adult drinking behaviors may show some systematic deviation from adult drinking behaviors in a given culture, however, because of differences in living conditions between adolescents/young adults and the adult population and because of the influences of international youth culture and mass media.

One source of information on alcohol consumption and its consequences in various areas of the world is the World Health Organization (WHO). For surveying purposes (e.g., to estimate the global burden of disease), WHO has divided the world into 15 geographical areas based on adult and infant mortality. WHO also has used these areas to estimate the levels of alcohol consumption and proportions of drinkers around the globe.^1^ However, countries within these 15 areas are not always uniform with regard to alcohol consumption and drinking habits. For example, Iceland and Norway, with a total alcohol consumption of about 7 liters per person age 15 and older, belong to the area “Europe A,” where the average alcohol consumption is 13 liters (i.e., almost twice that in Iceland or Norway). Despite these kinds of problems, the WHO data help to broadly characterize the levels and trends in alcohol consumption and drinking patterns in different parts of the world.

### General Trends in Total Alcohol Consumption

Rehm and colleagues (2003) conducted an international comparison of average alcohol consumption in people age 15 and older around the world using the WHO data. These analyses found the following:

- Average alcohol consumption was highest in Europe, the Americas, and established market economies such as Australia, Japan, and New Zealand, although there were exceptions (e.g., the Muslim countries of the former Soviet Union and Yugoslavia and the least developed countries in South and Central America).
- Average alcohol consumption generally was lower in Africa and Asia.
- Alcohol consumption was particularly low in the Muslim countries in the eastern Mediterranean region and on the Indian subcontinent.

Other studies have examined changes in per capita or per adult alcohol consumption since the mid-1970s (Babor et al. 2003) and have found that:

- Alcohol consumption appears to have declined in many countries with previously high alcohol consumption, particularly in the traditional wine-producing and wine-drinking countries of Europe (i.e., France, Italy, Portugal, and Spain) but also in the wine-producing countries of South America.
- In many other established market economies, such as Canada and the United States, a smaller but still significant decrease in total alcohol consumption has occurred over the same period.
- In most countries of the Americas and Africa and in the eastern Mediterranean countries, alcohol consumption has been constant or slightly decreasing during recent decades.
- Alcohol consumption appears to have increased the most in Asian countries.
- Some developed countries (e.g., Denmark, Finland, Ireland, and Japan) have countered the trend toward decreasing alcohol consumption; in fact, consumption there has increased.

### Proportions of Drinkers

The proportion of people who drink any alcohol varies greatly among different countries. In general, the highest proportion of drinkers is found in Europe, Australia, and New Zealand, where 80 to 90 percent of all adults are drinkers (Rehm et al. 2003). In the Western Pacific, 80 to 90 percent of all men are drinkers. In the Americas, about two-thirds of adults are drinkers. For instance, the share of drinkers is 73 percent in Canada and 65 percent in the United States (Babor et al. 2003). In African countries, around half of the men and one-third of the women drink alcohol. In the rest of the world, only a minority of adults are drinkers.

In all countries, men are more likely to drink alcohol than are women. The differences between men and women in the proportion of drinkers are particularly marked in China and Southeast Asia. Women are especially likely to be abstainers in the Indian subcontinent and Indonesia as well as in the Middle East (Rehm et al. 2003).

### Differences in Drinking Patterns

Many studies (primarily based on survey data) have analyzed drinking patterns around the world. These studies consistently have found significant differences in drinking patterns between men and women, between younger and older people, and often among ethnic or religious groups. For example, Ahlström and colleagues (2001*a*) found that, on average, men drink significantly more than women do. In many countries, men account for 70 to 80 percent or more of the total alcohol consumption, and in some developing countries, men’s share of overall alcohol consumption is even greater. For instance, survey data from China indicate that men consume about 95 percent of all alcohol (Babor et al. 2003).

Alcohol consumption also is unevenly distributed among the drinking population in any country—that is, in all societies, most of the alcohol is consumed by a relatively small proportion of drinkers. For example, in the United States, the top 20 percent of drinkers consume almost 90 percent of all alcohol (Greenfield and Rogers 1999). And in China, the top 12.5 percent of the drinkers (corresponding to 7.5 percent of the population) have been estimated to account for 60 percent of total alcohol consumption (Wei et al. 1999). In general, the proportion of drinkers who account for most of the alcohol consumption probably is smaller in countries with low per capita alcohol consumption—that is, in these countries alcohol consumption is more concentrated (Edwards et al. 1994).

### Intoxication and Harmful Drinking

Countries also vary in how often people drink to intoxication, how intoxicated people get, and how people behave while intoxicated. Generally, men are more likely than women to consume large quantities of alcoholic beverages or drink to intoxication (Babor et al. 2003). Also, the proportion of heavy drinkers and the frequency of heavy-drinking occasions are higher among men than among women. Consequently, patterns of harmful drinking^2^ are more common in men than in women. Some evidence suggests that this phenomenon may be even more pronounced in developing countries (Room et al. 2002).

According to WHO’s data on the global burden of disease, people in the former socialist countries of Eastern Europe, in Middle and South America, and in parts of Africa exhibit the most detrimental drinking patterns. For example, in these countries, drinking to intoxication is a characteristic mediator between alcohol consumption and alcohol-related morbidity, mortality, and social harms. Conversely, drinking patterns appear to be least detrimental in Western Europe, as represented by the patterns found in the wine-producing countries of southern Europe, where people primarily consume wine with meals and do not drink to intoxication (Rehm et al. 2003).

## Drinking Patterns of Adolescents and Young Adults

The drinking patterns of people in various age groups are difficult to compare internationally because population surveys conducted in different countries often use different age groupings and varying measures of alcohol consumption levels. Furthermore, most surveys that compare drinking in various age groups have been conducted in the established market economies of Europe and North America, and their findings do not always apply to other regions of the world. Nevertheless, two common findings have emerged from these studies (Babor et al. 2003): (1) abstinence is more prevalent in older age groups than among young adults, and (2) intoxication is more frequent among young adults than older people.

One survey that has provided basic information on adolescents’ alcohol consumption in various European countries is the European School Survey Project on Alcohol and Other Drugs (ESPAD) (Hibell et al. 2004). This multinational study of drinking habits and drug use among 15- to 16-year-old European students was first conducted in 1995, and a second survey followed in 1999. For the most recent data collection, conducted in 2003, students from 35 European countries filled out anonymous self-administered in-school questionnaires. Sample sizes, which were designed to be nationally representative, ranged from 555 in Greenland to almost 6,000 in Poland. The findings of this and other relevant surveys are summarized in the following sections.

### Age at Onset of Drinking and Prevalence of Abstinence

Both the long-term and short-term health effects of alcohol consumption depend at least in part on the age when the person begins to consume alcohol. For example, results from a U.S. survey indicate that compared with those who begin drinking at a later age, respondents who begin drinking in their teenage years are more likely during late adolescence and adulthood to experience alcohol-related unintentional injuries (e.g., motor vehicle crashes, falls, burns, and drownings) and to be involved in fights after drinking (Hingson et al. 2000). Furthermore, early onset of regular alcohol consumption is a significant predictor of lifetime alcohol-related problems, at least in some Western countries (Chou and Pickering 1992; Grant and Dawson 1997; Kraus et al. 2000; Pulkkinen et al. 2003). However, it is not clear whether starting to drink at an early age actually causes alcohol-related problems and alcohol use disorders or whether it simply indicates an existing vulnerability to alcohol use disorders (Dawson 2000).

In the United States, the average age of first-time use of alcohol is about 13 years. In contrast, in a survey conducted in 23 European countries in the late 1990s, more than half of 11-year-olds reported having tasted alcohol (Jernigan 2001). Still, at the beginning of adolescence, the abstinence rate in all European countries is high compared with the adult abstinence rate.

In the ESPAD study, the highest abstinence rate among European youths, 36 percent, was found in Iceland (table 1). In the other Nordic alcohol-monopoly countries Finland, Norway, and Sweden (i.e., former spirit-consuming countries^3^), about 20 percent of the 15- to 16-year-old students had not consumed any alcoholic beverages during the previous 12 months. Also, in some of the southern European wine-preferring countries, many of which are characterized as having the highest alcohol consumption in Europe, abstinence rates among youth were high (e.g., 26 percent in Portugal, 25 percent in Spain, and 20 percent in France) (see table 1). Conversely, the abstinence rate in Greece—also a wine-preferring country—was only 9 percent. Equally low or even lower abstinence rates also were found in beer-preferring countries such as Austria, the Czech Republic, Denmark, Germany, and the United Kingdom (Hibell et al. 2004). All of these abstinence rates are substantially lower than in the United States, where 41 percent of students reported not having consumed any alcoholic beverages during the last 12 months (Johnston et al. 2004).

Between adolescence and the onset of adulthood, abstinence rates decreased in most countries, and were about the same for people at age 25 as for middle-aged adults. Moreover, in many countries, differences in abstinence rates between males and females were smaller at age 15 than at age 25 (see Hibell et al. 2004; Rehm et al. 2001). In fact, among 15- to 16-year-old students, females in many countries showed lower abstinence rates than males (table 1).

### Lifetime Frequency of Drinking

The frequency of alcohol consumption among adolescents is still relatively low. In almost all ESPAD countries and in the United States, less than half of 15-to 16-year-old students were considered frequent drinkers—that is, they had consumed alcohol on 40 or more occasions during their lifetime (see table 2) (Hibell et al. 2004). The only country where 50 percent of adolescents reported such a frequency of alcohol consumption (i.e., were frequent drinkers) was Denmark. Otherwise, the countries with the highest proportion of frequent drinkers were Austria, the Czech Republic, the Netherlands, Ireland, and the United Kingdom. The lowest proportions of frequent drinkers were found in Greenland, Iceland, Norway, Portugal, and Turkey. In most countries, more boys than girls reported a lifetime prevalence of drinking at least 40 times.

### Drinking to Intoxication

In many cultures, drinking to intoxication is particularly characteristic of adolescents and young adults, and young males are more likely to drink to intoxication than young females (Currie et al. 2004; Hibell et al. 2004; Kuntsche et al. 2004).

According to the ESPAD study, it is not uncommon for students to drink to intoxication, although the prevalence of drunkenness varies considerably across the countries (see table 2). Thus, in the Nordic and Baltic countries as well as in Austria, the Czech Republic, Ireland, and the United Kingdom, nearly 20 percent or more of students reported having been drunk at least 20 times in their lives, compared with about 5 percent or less of students in most southern European countries and in Belgium and the Netherlands (Hibell et al. 2004). In other central European and in eastern European countries as well as in the United States, the proportions of students who reported having been drunk at least 20 times were intermediate.

### Frequency and Amount of Drinking

The ESPAD study also compared the number of drinking occasions and the amounts consumed per occasion by adolescents in the different countries. These analyses found that in the wine-producing countries (i.e., France, Greece, Italy, and Portugal), adolescents’ alcohol consumption can be characterized as fairly frequent but modest (Ahlström et al. 2001*b*). In the Nordic countries (i.e., Finland, Iceland, Norway, and Sweden), in contrast, alcohol consumption can be characterized as seldom but to intoxication. In the beer-preferring nations of Denmark, Ireland, and the United Kingdom, the students drink frequently and to intoxication (see the figure). However, this is not the case in all beer-preferring countries (e.g., Germany, the Czech Republic, and Belgium), demonstrating that drinking habits are not properties of alcoholic beverages and that certain kinds of beverages may be used in different ways. Nevertheless, the prevailing drinking patterns exhibit some relationship with the preferred alcoholic beverages.

### Time of Alcohol Consumption

One way to describe drinking patterns is to investigate how alcohol consumption is integrated into everyday activities (e.g., consumption with meals) (Ahlström-Laakso 1976). In many wine-producing countries, drinking is an integral part of meals. For instance, Italian adults rarely drink between meals. In contrast, in countries such as the United Kingdom, the United States, and the Nordic countries, most people drink at times other than meals.

The drinking patterns of adolescents and young adults in the various countries mirror those of the adults. Thus, the prevalence of intoxication (which typically results from alcohol consumption outside of meals) was much lower among adolescents in wine-producing European countries than among adolescents in Anglo-Saxon and Nordic countries (Currie et al. 2004; Hibell et al. 2004). In recent years, however, people in Mediterranean countries, especially young people, have begun to consume wine at times other than meals (Nahoum-Grappe 1995), and adolescents in these countries have begun to drink to intoxication more frequently. These observations suggest that moderate, controlled drinking in the wine-producing countries is being replaced by a more irregular pattern of drinking leading to inebriation (Grosso 2004). Moreover, drunken behavior has become more frequent on festive occasions. Thus, the drinking behavior of adolescents from wine-producing countries is beginning to resemble the behavior of adolescents from countries such as Denmark, Finland, and the United Kingdom.

### Consequences of Drinking

Most alcohol-related problems that affect young people do not result from chronic heavy drinking but from occasional heavy drinking and intoxication. Only a minority of young adults—most of them men—drink heavily on a regular basis, thereby increasing their risk of encountering health problems typically associated with chronic alcohol consumption (e.g., liver disease). However, even young people (including the majority of young women) who generally drink in moderation may occasionally drink heavily, thereby increasing their risk for certain adverse effects. For example, alcohol interferes with cognitive, perceptual, and motor skills and therefore can contribute to unintentional injuries and deaths, particularly after heavy alcohol consumption. In fact, in many societies, alcohol-related fatalities are particularly common among young adults and contribute substantially to alcohol-related mortality (Ahlström 2002). This particularly high mortality rate is attributable in part to the drinking patterns of young adults (i.e., drinking heavily or to intoxication) and in part to their lack of experience with and tolerance for alcohol, which may make them more likely, for example, to be involved in alcohol-related traffic crashes than more experienced drinkers (Ahlström 2002).

In the established market economies of Europe, the proportion of alcohol-related deaths is higher among young men than among young women. Among 15- to 29-year-olds, 12.8 percent of all deaths among males and 8.3 percent of all deaths among females have been estimated to be attributable to alcohol, indicating that alcohol-related deaths account for a considerable amount of mortality in young Europeans (Rehm et al. 2001). Traffic crashes are the main cause of alcohol-related deaths for both genders, followed by self-inflicted injuries; alcohol-related chronic diseases are not common causes of death in this age group. Both the average volume of alcohol consumption and patterns of drinking influence the rate of alcohol-related deaths.

Young adults are susceptible not only to alcohol-related health problems (i.e., deaths and injuries) but also to alcohol-related social problems. In fact, for any given level of drinking, young adults report more social problems than do middle-aged adults. These alcohol-related social problems include problems with family, friends, and at work; financial difficulties; legal problems, such as property damage, public disturbance, violence, or sexual assault; and other risk-taking behavior. The probability of young people suffering such social consequences increases with their level of drinking.

**Figure:**
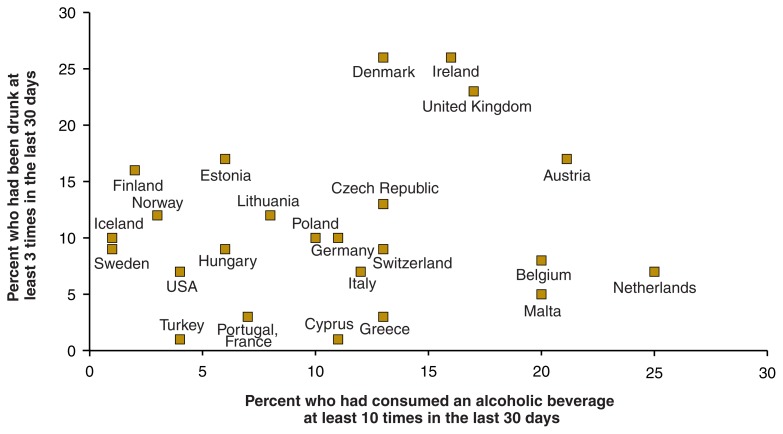
Frequency of drinking and of getting drunk during the last 30 days among European and American 15- and 16-year-olds, 2003. Data on European adolescents were taken from the 2003 European School Project on Alcohol and Other Drugs (ESPAD). SOURCE: Hibell et al. 2004.

## Factors Affecting Drinking by Adolescents and Young Adults

In general, a person’s drinking behavior is affected by two groups of factors (Quigley and Marlatt 1996; also see Jackson et al. 2005):

- Internal factors, such as gender, personality factors, and biological traits (e.g., certain genetic predispositions).
- External factors, such as social norms and the physical availability and prices of alcoholic beverages.

(For more information on genetic factors that influence drinking behavior, see the article by Rose in this issue. For discussion of social and other environmental factors that influence alcohol consumption by young adults, see the articles by White and Wagenaar and colleagues.)

### Gender Differences

Gender and age are important factors affecting drinking behavior. In almost every society, young adult males drink more often than young adult females. During adolescence, drinking patterns are less differentiated by gender, and at the onset of young adulthood, females may even drink more frequently than males, partly because females typically mature earlier than males and partly because they do not yet have family bonds and responsibilities. Once they reach and pass through young adulthood, however, young women tend to consume less alcohol, drink less frequently, and get drunk less often than young men (Ahlström et al. 2001*a*).

The ESPAD study as well as some other analyses have demonstrated that many countries now show a convergence of boys’ and girls’ drinking patterns that blurs the distinction between the genders (Currie et al. 2004; Hibell et al. 2000, 2004; Johnston et al. 2000). For example, although consumption of beer and spirits still is more frequent among boys than girls in most ESPAD countries, prevalence rates for consumption of spirits are equal or almost equal between the sexes in nearly half of the countries. Likewise, the frequency of intoxication is similar for both genders in many ESPAD countries (Hibell et al. 2004). The convergence of drinking patterns is especially obvious in the Nordic countries, Ireland, the United Kingdom, and the United States. In the eastern and southern European countries, however, large gender differences still exist.

### Social Norms and Other Social Factors

Social norms about when, how often, and at what level drinking is considered acceptable vary among different countries or even among population subgroups within a country, and they may change over time. Other social factors, such as the context of the drinking occasion, also can influence people’s drinking behavior. In a Dutch study investigating the factors contributing to young adults’ heavy drinking in public during weekends, social norms condoning alcohol consumption and drinking occasions characterized by socializing were identified as the most important factors (Oostveen et al. 1996). Modeling was the third most influential variable, but direct social pressure had only minimal influence.

Thus, it appears that for young adults, drinking not only is a matter of personal experimentation but also can reflect a desire to fit in with peers and others (Ahlström 2000). Consistent with this hypothesis, evidence suggests that the most reliable predictor of adolescents’ drinking behavior is the drinking behavior of their friends, followed by the drinking behavior of their siblings. Other family factors—such as parent–child relationships, communication, and parenting practices—also significantly influence the drinking behavior of adolescents (Ahlström 2002).

Considerable evidence suggests that drinking patterns for all adults also depend on education, social class, occupation, employment status, place of residence, race or ethnicity, and religion. However, few studies have investigated the influence of these factors on young adults. One comparative study conducted in nine European countries examined the influence of marriage, parenthood, and employment on drinking behavior. These transitions tend to occur during early adulthood and demand the adoption of new social roles. The study found that parenthood was profoundly and consistently associated with women’s drinking patterns—that is, women who had children, particularly young mothers, consumed less alcohol than did women without children (Ahlström et al. 2001*a*). In the United States, religion also is an important factor affecting the age and onset of drinking. The importance of religion is further highlighted by the low alcohol consumption levels and high rates of abstinence in Muslim countries.

The influence of any social factor can differ profoundly among various countries. For example, in some countries a person’s place of residence (e.g., a rural or urban area) has been shown to influence what type of beverage he or she primarily consumes. In wine-producing countries wine consumption is usually more popular in rural areas, but in the Nordic countries wine is consumed primarily in urban areas. This is explained for the most part by the fact that in wine-producing countries wine is a traditional drink with meals, whereas in the Nordic countries wine drinking is associated with modern living styles (Simpura and Karlsson 2001).

### Alcohol Availability and Pricing

Most countries have a minimum legal drinking age (MLDA) for purchasing, selling, or consuming alcoholic beverages. In most European countries, the MLDA is 18 years or less (see table 3). Several other countries—such as the United States, Egypt, Indonesia, Micronesia, Palau, Samoa, and the Solomon Islands—do not allow people to purchase, sell, or consume alcoholic beverages before the age of 21 (Jernigan 2001; WHO 2004). In many countries, the MLDA is the same for all alcoholic beverages, but in countries that have different age limits for different beverage types, the age limits usually are lower for beer and wine than for distilled beverages (Österberg and Karlsson 2002). In most countries, MLDAs are the same for off-premise and on-premise sales.

Alcohol taxation and high alcohol prices as well as strict regulation of physical alcohol availability (i.e., sale of alcoholic beverages only in limited locations or at certain times) are powerful policy tools for controlling alcohol consumption (Babor et al. 2003). Although such measures affect the entire population, some studies have found that high alcohol prices particularly affect adolescents and young adults, who in many societies have fewer economic resources than older adults (Williams et al. 2005).

Evidence also suggests that the amount of money available to people influences the frequency of drinking. For example, Rahkonen and Ahlström (1989) analyzed trends in drinking habits among Finnish adolescents between 1973 and 1987. They found that drinking frequency decreased at the beginning of the 1980s but then began to increase by 1983. This rise was explained by increased alcohol availability and by an increase in money available to adolescents for their leisure time activities. Similar results were obtained in a later study among Finnish students (Lintonen et al. 2000).

To assess the effectiveness of measures restricting alcohol availability in limiting alcohol consumption by adolescents, Grossman and colleagues (1995) compared the effects of alcohol pricing policies and changes in MLDA. These investigators demonstrated that both types of measures impacted alcohol use and mortality from alcohol-related motor vehicle crashes, although higher taxes on alcohol were more effective at reducing adolescent drinking than implementation of a uniform MLDA of 21.

## Summary and Discussion

Alcohol consumption is a social behavior, something people learn from and practice with other members of their culture (family, peers, etc.). Consequently, the drinking behavior of adolescents and young adults in any country or culture is related to the drinking behavior of the whole population. International studies have demonstrated that adult drinking patterns vary greatly among different countries and cultures over time and between different population groups within a given country. Speaking very generally, the frequency and amount of alcohol consumption are highest in Europe, North America, and other countries with established market economies; lower in Africa and Asia; and particularly low in the Muslim countries of the eastern Mediterranean region and the Indian subcontinent. In recent decades, alcohol consumption has decreased especially in southern Europe and increased in Asia.

Despite its similarities to adult drinking, alcohol consumption by adolescents and young adults has some special characteristics because the way of life and living conditions of 14- to 25-year-olds in any culture differ somewhat from those of older adults. For example, the mass media (particularly the advertising industry), the Internet, and international youth cultures also affect the drinking patterns of adolescents and young adults (Unger et al. 2003; Carroll and Donovan 2002).

One difference between adolescent/young adult drinking patterns and adult drinking patterns concerns gender differences. In almost all cultures, men abstain less frequently than women and drink more frequently and in greater quantities than women. Among adolescents and young adults, especially at the onset of adolescence, however, these gender differences are less prominent or do not exist at all. Moreover, in many cultures, drinking to intoxication is more characteristic of adolescent and young adult drinking than of drinking by older adults. Finally, most of the alcohol-related problems that affect adolescents result from periodic heavy drinking and intoxication rather than from chronic alcohol consumption, because relatively few adolescents drink heavily on a regular basis. In contrast, older adults more frequently experience the adverse health effects (e.g., liver disease) that result from long-term alcohol consumption.

Because most of the information researchers have acquired about the drinking patterns of adolescents and young adults is based on data obtained in European and North American countries, global comparisons of drinking patterns are difficult to make. In addition, less information is available on young adults than on adolescents, who, through school-based surveys, can be accessed more easily. To address these limitations and to allow for truly global and reliable comparisons of drinking patterns in adolescents and young adults, future studies should focus more on regions outside North America and Europe. Such analyses also could reveal characteristic patterns in the factors affecting adolescent and young adult drinking behavior. Finally, studies following participants over time (i.e., longitudinal studies) are needed to better evaluate the findings of one-time cross-sectional studies.

The generalizability of existing research on the effects of alcohol pricing on adolescent and young adult drinking is limited because most of these studies have been conducted in the United States. Recent price decreases in Europe, especially in the Nordic countries, however, will provide researchers with an opportunity to study whether changes in price particularly affect alcohol consumption by adolescents and young adults in other countries as well.

## Figures and Tables

**Table 1:** Abstinence Rates Among European and American 15- and 16-Year-Olds During the Last 12 Months*

Country	All Students (%)	Males (%)	Females (%)
Austria	7	8	6
Belgium	14	13	15
Bulgaria	14	13	14
Croatia	18	15	21
Cyprus	21	16	26
Czech Republic	5	5	5
Denmark	5	4	5
Estonia	13	14	11
Faroe Islands	24	24	24
Finland	20	22	19
France	20	18	22
Germany	7	7	7
Greece	9	7	10
Greenland	27	32	23
Hungary	16	16	16
Iceland	36	38	35
Ireland	12	14	10
Isle of Man	6	8	4
Italy	18	15	20
Latvia	13	14	12
Lithuania	6	6	6
Malta	10	9	11
Netherlands	15	14	15
Norway	24	26	21
Poland	15	12	17
Portugal	26	24	28
Romania	20	16	23
Russia (Moscow)	14	18	11
Slovak Republic	10	10	9
Slovenia	17	15	19
Spain	25	26	24
Sweden	23	23	23
Switzerland	12	12	13
Turkey	65	60	72
Ukraine	16	17	15
United Kingdom	9	10	8
United States	41	43	39

* Data on European adolescents was taken from the 2003 European School Project on Alcohol and Other Drugs (ESPAD).

SOURCE: Hibell et al. 2004.

**Table 2:** Prevalence of Various Indicators of Drinking Behavior Among European and American 15- and 16-Year-Olds*

Country	Lifetime Alcohol Consumption	Intoxication		Binge Drinking
		
	Percent who had consumed alcohol at least 40 times in their lives	Percent who had been intoxicated at least 20 times in their lives	Percent who had been intoxicated at least 3 times in the past 30 days	Percent who had consumed at least 5 drinks in a row at least 3 times in the past 30 days
Austria	48	21	17	NA**
Belgium	36	7	8	22
Bulgaria	27	10	10	21
Croatia	27	9	8	15
Cyprus	21	2	1	10
Czech Republic	46	18	13	18
Denmark	50	36	26	24
Estonia	32	26	17	20
Faroe Islands	32	24	18	19
Finland	20	26	16	15
France	22	3	3	9
Germany	37	12	10	28
Greece	35	3	3	11
Greenland	13	21	19	19
Hungary	21	11	9	8
Iceland	14	16	10	11
Ireland	39	30	26	32
Isle of Man	45	29	23	27
Italy	24	5	7	13
Latvia	26	14	8	22
Lithuania	38	21	12	13
Malta	33	4	5	25
Netherlands	45	6	7	28
Norway	15	14	12	24
Poland	27	10	10	11
Portugal	14	3	3	16
Romania	18	3	3	11
Russia	39	15	11	17
Slovak Republic	34	14	11	15
Slovenia	25	15	12	22
Sweden	17	17	9	25
Switzerland	27	10	9	15
Turkey	7	1	1	5
Ukraine	22	18	16	22
United Kingdom	43	27	23	27
United States	12	7	7	9

* Data on European adolescents was taken from the 2003 European School Project on Alcohol and Other Drugs (ESPAD).

** NA = no data available

Source: Hibell et al. 2004.

**Table 3:** Minimum Legal Drinking Ages (MLDAs) for Off-Premise and On-Premise Sales of Various Types of Alcoholic Beverages in European Countries

Country	Off-Premise			On-Premise		
	
	Beer	Wine	Spirits	Beer	Wine	Spirits
Austria	16	16	18	16	16	18
Belgium	—*	—	18	16	16	18
Bulgaria	18	18	18	18	18	18
Cyprus	NA**	NA	NA	NA	NA	NA
Czech Republic	18	18	18	18	18	18
Denmark	16	16	16	18	18	18
Estonia	18	18	18	18	18	18
Finland	18	18	20	18	18	18
France	16	16	16	16	16	16
Germany	16	16	18	16	16	18
Greece	—	—	18	—	—	18
Hungary	18	18	18	18	18	18
Iceland	20	20	20	20	20	20
Ireland	18	18	18	18	18	18
Italy	16	16	16	16	16	16
Latvia	18	18	18	18	18	18
Lithuania	18	18	18	18	18	18
Luxembourg	—	—	—	18	18	18
Malta	16	16	16	—	—	—
Netherlands	16	16	18	16	16	18
Norway	18	18	20	18	18	20
Poland	18	18	18	18	18	18
Portugal	16	16	16	16	16	16
Romania	18	18	18	18	18	18
Slovak Republic	18	18	18	18	18	18
Slovenia	15	15	15	15	15	15
Spain	16	16	16	16	16	16
Sweden	18	20	20	18	18	18
Switzerland	16	16	18	16	16	18
Turkey	18	18	18	18	18	18
United Kingdom	18	18	18	16	16	18

* — = no legal age limit

** NA = no data available

SOURCE: WHO 2004.

## Footnotes

1 These data are derived primarily from population-weighted averages of country estimates, but in many cases the figures include a substantial element of extrapolation and expert judgment (Babor et al. 2003).

2 Harmful drinking refers to any drinking pattern where drinking causes immediate harm to the drinker or others, including physical harm (e.g., resulting from alcohol-related unintended injuries and car crashes), social harm (e.g., arguments with family members or others), economic harm (e.g., lost property), or legal harm (e.g., conviction for drunk driving).

3 Although some decades ago there seemed to be relatively clear distinctions among European countries with respect to the beverages of preference—that is, northern European countries were classified as spirit-consuming, Anglo-Saxon and Central European countries were considered beer-consuming, and southern European countries were considered wine-consuming—drinking patterns in many countries have changed, and these distinctions have been blurring in recent decades.
